# Advancing the Understanding of Environmental Transformations, Bioavailability and Effects of Nanomaterials, an International US Environmental Protection Agency—UK Environmental Nanoscience Initiative Joint Program

**DOI:** 10.4236/jep.2018.94025

**Published:** 2018-04-02

**Authors:** Mitch M. Lasat, Kian Fan Chung, Jamie Lead, Steve McGrath, Richard J. Owen, Sophie Rocks, Jason Unrine, Junfeng Zhang

**Affiliations:** 1 Office of Research and Development, United States Environmental Protection Agency, Washington DC, USA; 2 National Heart and Lung Institute, Imperial College, London, UK; 3 Centre for Environmental Nanoscience and Risk, University of South Carolina, Columbia, USA; 4 University of Birmingham, Edgbaston, UK; 5 Rothamsted Research, Harpenden, UK; 6 Business School, University of Exeter, Exeter, UK; 7 Institute for Resilient Futures, Cranfield University, Cranfield, UK; 8 Department of Plant and Soil Sciences, University of Kentucky, Lexington, USA; 9 Nicholas School of the Environment, Duke University, Durham, USA

**Keywords:** Aquatic Environment, Consumer Products, Manufactured Nanomaterials, Predictive Models, Terrestrial Ecosystem

## Abstract

Nanotechnology has significant economic, health, and environmental benefits, including renewable energy and innovative environmental solutions. Manufactured nanoparticles have been incorporated into new materials and products because of their novel or enhanced properties. These very same properties also have prompted concerns about the potential environmental and human health hazard and risk posed by the manufactured nanomaterials. Appropriate risk management responses require the development of models capable of predicting the environmental and human health effects of the nanomaterials. Development of predictive models has been hampered by a lack of information concerning the environmental fate, behavior and effects of manufactured nanoparticles. The United Kingdom (UK) Environmental Nanoscience Initiative and the United States (US) Environmental Protection Agency have developed an international research program to enhance the knowledgebase and develop risk-predicting models for manufactured nanoparticles. Here we report selected highlights of the program as it sought to maximize the complementary strengths of the transatlantic scientific communities by funding three integrated US-UK consortia to investigate the transformation of these nanoparticles in terrestrial, aquatic, and atmospheric environment. Research results demonstrate there is a functional relationship between the physicochemical properties of environmentally transformed nanomaterials and their effects and that this relationship is amenable to modeling. In addition, the joint transatlantic program has allowed the leveraging of additional funding, promoting transboundary scientific collaboration.

## 1. Introduction

Emerging results have indicated nanotechnology has the potential to impact industrial processes (e.g., magnetic storage applications and catalysis), create materials with superior properties, and improve the effectiveness of drug delivery. Nanomaterials also offer detection advantages for use in national security emergencies, and innovative approaches to address current environmental concerns [1] [2] [3]. Because of its promise, nanotechnology has been widely heralded as the underpinning technology platform of the next industrial revolution [4] and its development was deemed essential for economic development and United States (US)national se curity (https://clintonwhitehouse4.archives.gov/textonly/WH/EOP/OSTP/html/00_121_3.html).

In 2000, the National Nanotechnology Initiative (NNI) was created in the US to ensure the development of nanotechnology (https://clintonwhitehouse4.archives.gov/textonly/WH/EOP/OSTP/html/00_121_3.html). At the foundation of the Initiative was the vision of enabling the control of matter at the nanoscale in order to facilitate a revolution in technology and industry. To materialize this vision, the US Congress enacted the 21st Century Nanotechnology Research and Development Act (P.L. 108–153). The Act provided a statutory foundation for the NNI, established programs, assigned federal agency responsibilities, authorized funding levels, and promoted nanotechnology research to address key issues. To ensure harmonious progress of the Initiative, federal agencies were tasked with developing programs consistent with their mission and authority. Under the NNI guidance, the Office of Research and Development (ORD) in the Environmental Protection Agency (EPA), has initially developed a program of research grants focused on environmental applications of nanotechnology with an emphasis on prevention, detection, and remediation of environmental pollution. As concerns of the unintended consequences of nanotechnology have emerged, the focus of EPA’s nanotechnology grants program shifted to advance the understanding of the environmental implications and risks of nanotechnology.

In 2004, the Royal Society and Royal Academy of Engineering in the United Kingdom (UK) published a seminal report highlighting how little it was known about how manufactured nanomaterials enter the environment, how they behave, their fate, and possible effects on plants, animals and humans (https://royalsociety.org/~/media/Royal_Society_Content/policy/publications/2004/9693.pdf). The 2005 UK Government response highlighted the need for a cross-disciplinary approach led by the research community (http://webarchive.nationalarchives.gov.uk/20070603164510/http://www.dti.gov.uk/files/file14873.pdf). To address the challenge, in 2006, in the UK a group of funding agencies led by the Natural Environment Research Council (NERC) established the Environmental Nanoscience Initiative (ENI)^1^, a research program on hazard, exposure and environmental risk posed by manufactured nanomaterials. ENI funded exploratory research projects in 2006 and 2007 aimed at building capacity within the UK scientific community. At the same time, the increasing awareness about the knowledge gaps surrounding environmental implications of nanotechnology was highlighted in a report by the Royal Commission on Environmental Pollution published in 2008 (https://www.gov.uk/government/uploads/system/uploads/attachment_data/file/228871/7468.pdf). Again, the UK Government response agreed with the report’s recommendations as to the need to create an integrated approach to investigate the environmental implications of nanotechnologies with a priority focus on commercially available nanomaterials (https://www.gov.uk/government/uploads/system/uploads/attachment_data/file/228785/7620.pdf). These recommendations strengthen the ENI vision to consider large research projects of transdisciplinary nature joining expertise from across continents.

Rapid development and use of manufactured nanomaterials (MNMs) has led to concerns about their exposure and hazard [5] and underpinning environmental, health and safety research should aim to allay those concerns through appropriate scientific means. Better science should lead to action to protect human and environmental health and promote the benefits of nanotechnology in a sustainable manner. Understanding the risks posed by MNMs is a global challenge that is best addressed through international collaboration and multidisciplinary expertise to allow resource and knowledge sharing. By 2009, the USEPA/ORD and UKENI had developed strong research communities through their nanotechnology grants programs and the benefits of joining these communities to collaboratively address the potential implications of nanotechnology became readily apparent. Initial consultations with the research community and other stakeholders indicated that one major goal for an international collaboration is the development of predictive models of fate, behavior, bioaccumulation, and effects of MNMs through relevant pathways of exposure via water, air, and land.

In 2009, The UKENI and USEPA/ORD’s Science to Achieve Results (STAR) grants program (https://www.epa.gov/research-grants) announced the development of a bilateral nanotechnology research program (https://cfpub.epa.gov/ncer_abstracts/index.cfm/fuseaction/display.rfatext/rfa_id/516; http://www.nerc.ac.uk/research/funded/programmes/nanoscience/ao-eni2/) with financial support provided jointly by UKENI, USEPA and the US Consumer Product Safety Commission (USCPSC). The program issued a call for proposals and subsequently funded three large, interdisciplinary UK-US consortia; 1) Transatlantic Initiative for the Nanotechnology and the Environment (TINE), 2) Manufactured Nanomaterial Bioavailability & Environmental Exposure (Nano-Bee) and 3) Risk Assessment for Manufactured Nanoparticles Used in Consumer Products (RAMNUC). Each consortium developed an integrated, transatlantic team of scientists, with complimentary expertise to address joint research objectives. The research objectives, specific media, and case studies associated with these consortia are shown in Table 1.

## 2. Results

### 2.1. Transatlantic Initiative for Nanotechnology and the Environment (TINE) Consortium, MNMs in the Terrestrial Ecosystems

The primary route for MNMs (e.g., used in consumer products) to enter terrestrial ecosystems is by wastewater treatment plants [6] via the land application of biosolids (sludge) to enhance soil fertility [7]. Previous studies have focused on pristine synthesized nanomaterials [8] which may misrepresent MNMs realistic environmental exposures [9] [10]. In contrast, TINE consortium sought to characterize transformation of zinc oxide (ZnO), titanium dioxide (TiO_2_) and silver (Ag) nanoparticles in realistic environmental conditions by investigating their behavior, bioavailability, trophic transfer, and ecological effects during sewage treatment and following application to agricultural soils. TINE also aimed to determine whether risk assessment models and regulations for land applications of biosolids could be extrapolated to MNMs.

**Figure 1:**
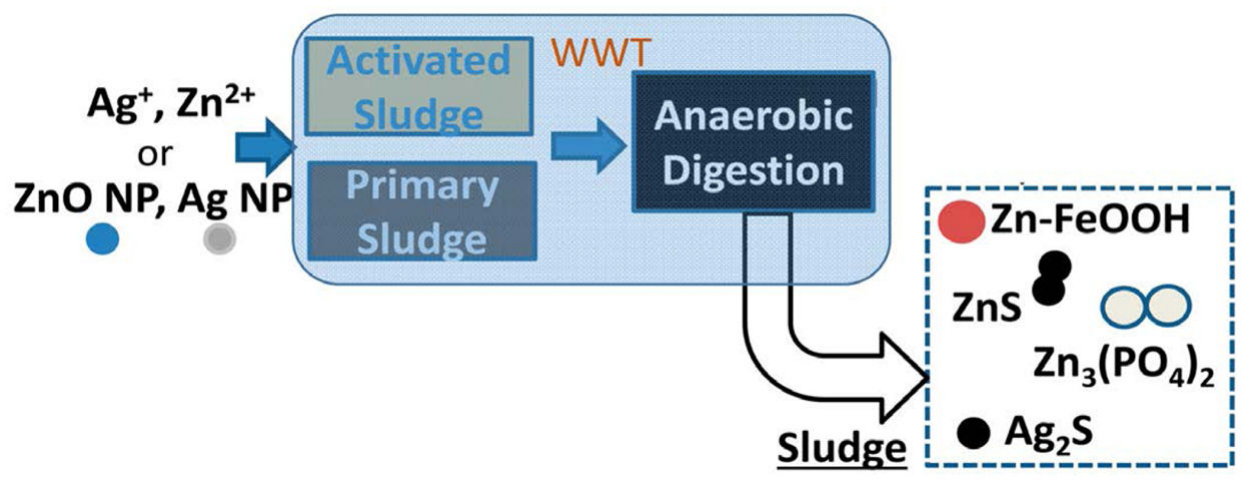
Similar transformation of disolved, bulk or nanosized metals (ZnO and Ag) in the wastewater treatment plant (WWTP). X-ray absorption spectroscopy revealed that disolved, bulk or nanosized ZnO and Ag particles are transformed to sulfide and phosphate minerals. Zn was also bound to iron oxohydroxides. Reprinted with permission from Ma *et al*., 2014. Copyright 2014, American Chemical Society.

Pilot-scale wastewater treatment plant studies showed that nanomaterials mostly partitioned in the sewage sludge with only a low fraction recovered in the treatment process effluents. Within the treatment plant, Ag and ZnO nanomaterials were completely transformed to a variety of secondary mineral phases. According to X-ray based speciation studies, transformation of bulk (nanomaterial free) was similar to that of dissolved metals (Figure 1). Laboratory assays, conducted to determine the type and range of transformations, predicted well the transformations that occurred in the wastewater treatment plant [11] [12] [13] [14] [15]. In laboratory-scale experiments, researchers found that the type of particle coating affected the nanomaterial binding to soil solids, but not in aged nanomaterials which behaved identically irrespective of the original coating [16]. These results demonstrate that the transformations during wastewater treatment can alter the influence of initial particle coating on nanomaterial behavior. This observation could greatly simplify the risk assessment process, since one major material variable (particle coating) may have little influence on nanoparticles behavior in soils when applied as biosolids.

Xray spectroscopy-based speciation and bulk extractions results did not predict differences in the bioavailability and biological responses between bulk/dissolved metals and nanoparticles. Studies with plants showed that the legume *Medicago trunculata*g rew less, had fewer root nodules, and accumulated more Zn when exposed to aged nanomaterial-treated biosolids compared with the bulk-treated biosolids [17]. In addition, patterns of gene expression and the behavior of the soil microbial community were significantly altered by the nanomaterial-treated biosolids [18]. These results indicate that current risk assessment procedures for land application of biosolids may need to be adjusted to account for differences in the behavior and toxicity of nanomaterials relative to dissolved metals.

In ecotoxicological studies, earthworms exposed to nanomaterial-treated bio-solids reproduced less than earthworms in bulk-treated biosolids. This effect was more likely caused by the exposure to Zn, in the biosolids, rather than Ag and TiO_2_ [19]. Transformed Ag and ZnO nanomaterials were considerably less toxic to nematodes than pristine nanomaterials and the effects appeared to be caused by a different mechanism of toxicity [20] [21] [22]. Therefore, ecotoxicity tests using pristine nanomaterials may not accurately predict the toxicity of nanomaterials following their transformation in the environment.

The research findings have been used to develop a first generation “Life-Cycle-Analysis-inspired Risk Assessment” (LCA-RA) predictive model of nanomaterials fate following transfer from sewage treatment plants to terrestrial environment. The TINE consortium developed a model of Ag and ZnO nanomaterials transport and transformation in agricultural fields and through watersheds [23] [24] [25] [26]. This realistic model accounts for the influence of land-use pattern, topography, meteorology, and stream hydrology on the transport and fate of nanomaterials. Researchers also developed a Bayesian risk forecasting model to predict the toxicity of Ag nanomaterials. The model can be easily adapted and updated as additional experimental data and other information on nanomaterial behavior in the environment become available. The baseline model suggests Ag nanoparticles may pose the greatest risk due to accumulation in aquatic sediments [27][28].

TINE researchers have also developed a new functional assay-based approach for predicting nanomaterial fate and effects [29] [30]. For this approach, the team has developed predictive parameters of mobility, bioavailability and toxicity by integrating intrinsic and extrinsic properties of nanomaterials within specific environmental matrices [31][32].

### 2.2. Manufactured Nanomaterial Bioavailability & Environmental Exposure Consortium (NanoBee), MNMs in the Aquatic Environment

The NanoBee consortium studied the exposure, bioavailability and toxicity of several nanoparticles including Ag, ceria (CeO_2_), ZnO, and Au in model aquatic organisms using a wide range of endpoints. Although some work was performed with commercial nanomaterials, a critical output was the generation of a library of nanomaterials with tightly controlled physical and chemical properties, which were characterized using a multi-method approach [33] [34]. The consortium developed isotopically-labelled nanomaterials [35] [36] to track nanomaterial movement through the ecosystemat very low concentrations. Additional work produced isotopically labelled nanohybrid tools to quantify the relative importance of ion and particle bioavailability and hence their toxicity [37]. Collaborative work between NanoBee and TINE researchers investigated and discussed properties of the pristine and transformed nanomaterials, determined their bio-availability and toxicological properties [38] and extensive characterization was conducted to quantify transformations and to understand the effect on uptake [39]. Physicochemical properties of environmentally transformed MNMs determined exposure concentrations and chemical speciation and were found to be essential in understanding MNMs bioavailability and toxicity.

Bioavailability was examined in experimental and modeling studies in *Lymnaea stagnalis*, *Daphnia magna* and *Lumbriculus variegatus*, with a particular focus on Ag nanoparticles [35] [40] [41] although other nanomaterials were also investigated [42]. In several studies, bioavailability and toxicity were examined together [43] with bioaccumulation studies suggesting that both the particle and the ion were bioavailable after dissolution although with different uptake and loss rate constants [35]. In a simplified freshwater food chain model comprising the green alga *Chlorella vulgaris* and the crustacean *Daphnia magna*, Ag nanoparticles had lower uptake rates than the dissolved Ag and showed a corresponding reduction in toxicity. In general, higher uptake values in the alga were related to higher toxicity, and electron microscopy showed the presence of Ag nanoparticles in the alga when exposed to higher nanoparticle concentrations. Additional studies suggested that toxicity was correlated with the ion concentration after dissolution [44]. A similar result was obtained in complementary genomic studies with zebrafish embryos [45] [46]. Thus, NanoBee research confirmed that Ag nanoparticle toxicity is largely, but not entirely, due to the ion which directly interacts with physiologically-active biological sites. It is likely that nanomaterials are an important delivery vehicle of toxic ionic species. However, not all toxicity could be explained by the ionic effect and this hypothesis needs further investigation. The observed uncertainties may be due to a current lack of appropriate experimental techniques, suggesting the need for new methodological developments, which are being developed from work started by NanoBee[37] [47].

The NanoBee consortium also studied the visualization of nanomaterials including Au, Ag and carbon-based nanomaterials which are difficult to visualize [48]. Researchers developed new methods for obtaining tightly constrained Au nanoclusters as internal standards for use in electron cryotomography. This led to the development of accurate, minimally-invasive 3D tools for visualizing nanomaterials in complex hydrated and organic media, and for studying nanomaterials interactions with proteins and other biological matrices [49]. The tool has led to the possibility of producing nanoscale images of easily perturbed biological structures such as proteins and eco-corona around nanomaterials [50]. Laboratory studies were conducted using appropriate modeling parameters (e.g., diffusion and sedimentation) and exposure media to simulate realistic environmental conditions [39]. Results suggested that nanomaterials are prone to a range of transformation processes such as dissolution, aggregation, eco-corona formation and sulfidation and that these effects are dependent on the solution and nanomaterial properties [51] [52]. For instance, a high resolution Scanning Transmission Electron Microscopy and Electron Energy Loss Spectroscopy (STEM-EELS) study showed heterogeneous sulfidation and allowed the quantification of the thickness of eco-coronas formed by natural organic macromolecules on nanomaterials [50] [53]. These results were linked with uptake and toxicity studies, suggesting that dispersed nanomaterials were far more toxic than aggregated nanomaterials [53] [54]; this work effectively complemented the Ag nanomaterial sulfidation work performed by TINE.

Nanoparticles toxicity was further studied in a range of aquatic organisms using both targeted toxicological assays and non-targeted (transcriptomic and metabolomics) approaches. At low concentrations, stimulatory effects have been seen in bacteria and hydroponically grown plants and toxicity was observed at higher concentrations [55] [56] [57]. Although extrapolation should be used cautiously, there is evidence of a potential hormetic effects. This assessment is further complicated by the concentration dependence of transformations [37] [50].

### 2.3. Risk Assessment for Manufactured Nanoparticles Used in Consumer Products Consortium (RAMNUC), MNMs in the Atmospheric and Indoor Air

The RAMNUC consortium conducted research to assess human health risk caused by inhalation exposure to MNMs (ZnO, Ag, and CeO_2_) incorporated in selected consumer products. The overall hypothesis of the RAMNUC consortium is that the physicochemical and toxicological properties of MNMs at the point of exposure will substantially differ from those at the source (synthesized in the laboratory or acquired commercially). These differences may have significant consequences on MNMs’ bioavailability, induction of oxidative stress, inflammation, and other toxicity measures. Therefore, RAMNUC aimed to understand how these nanoparticles transform as they enter and move through the atmosphere and become inhaled.

Exposure to airborne particles, ranging from 14 nm to 20 μm, resulting from the use of nanotechnology-based cosmetic powders, was studied by applying the aerosols to a mannequin’s face and measuring the concentration and size distribution of inhaled aerosol particles [58] [59]. The highest inhaled particle mass was in the coarse aerosol fraction (2.5 – 10 μm), while nanoscale particles were minimally inhaled. For all powders, 85% – 93% of aerosol deposition occurred in the head airways, while <10% deposited in the alveolar and <5% in the tracheo-bronchial regions with nanomaterials likely distributed as agglomerates. These results suggest a major nanomaterial deposition in respiratory system components other than alveolar regions and possibly a limited ability of the particles to enter the blood stream [60][61].

In cells isolated from human lung tissue, nanoparticles tested showed different bioactivity. In addition, RAMNUC results showed the lung lining fluid, and particularly dipalmitoylphosphatidylcholine, can modify the kinetics of ion release from nanoparticles. In human alveolar cells, Ag nanowires were dissolved and subsequently transformed into the highly insoluble Ag sulfide [62] [63]. Pulmonary surfactant also can significantly alter the dissolution kinetics, aggregation state and surface chemistry of ZnO nanowires (ZnONWs), with important consequences on how they are internalized and processed by the underlying epithelial cells [64]. *In vitro* adsorption of pulmonary surfactant lipids on ZnONWs has been demonstrated for the first time [65]. The lipid corona delayed the kinetics of Zn^2+^ release from ZnONWs at acidic pH, by blocking direct contact between the nanowire surface and the aqueous environment. In addition, pulmonary surfactant prevented the agglomeration of ZnONWs, possibly through contributions of both steric and charge stabilization effects. These results indicate a central role of pulmonary surfactant in understanding interactions at the bio-nano interface of the alveoli, and their impact on subsequent epithelial–endothelial nanoparticle translocation.

Exposure of lung cells to commercially available antifungal sprays containing nanoparticulate Ag (MesoSilver^™^ and Nanofix^™^) suggested an inflammatory response due to either product solvent and/or interaction of the Ag with the solvent. Similar results were reported with nanoparticulate Zn-containing sprays (TheraZinc^™^ and DermaZinc^™^). The overall toxicity was dependent on the type of nanoparticle and the solvent. These studies emphasize the importance of testing nanomaterials as they occur in commercial products, as the product solvent may alter the fate, behaviour and toxicity of the nanoparticles, and unintended inhalation could cause adverse health effects [66].

In another set of experiments, RAMNUC examined the potential toxic effects of Ag nanoparticles inhaled via the nasal passage in two rat strains (*i.e*., Brown-Norway and Sprague-Dawley). In the Brown-Norway rat strain, it was found that a pre-existing inflammatory condition is likely to lead to an increased amount of Ag retained in the lungs resulting in parenchymal dysfunction. This was not observed however in the Sprague-Dawley strain [67] [68]. The reduced clearance rate observed in the Brown-Norway rat may cause an increased inflammatory response, induction of surfactant protein D and phospholipids, and airway and parenchymal dysfunction. This would indicate that inhalation of Ag nanoparticles in humans with pre-existing lung condition (e.g., asthma or chronic obstructive pulmonary disease) may lead to a greater degree of inflammation with consequences on lung function.

**Figure 2:**
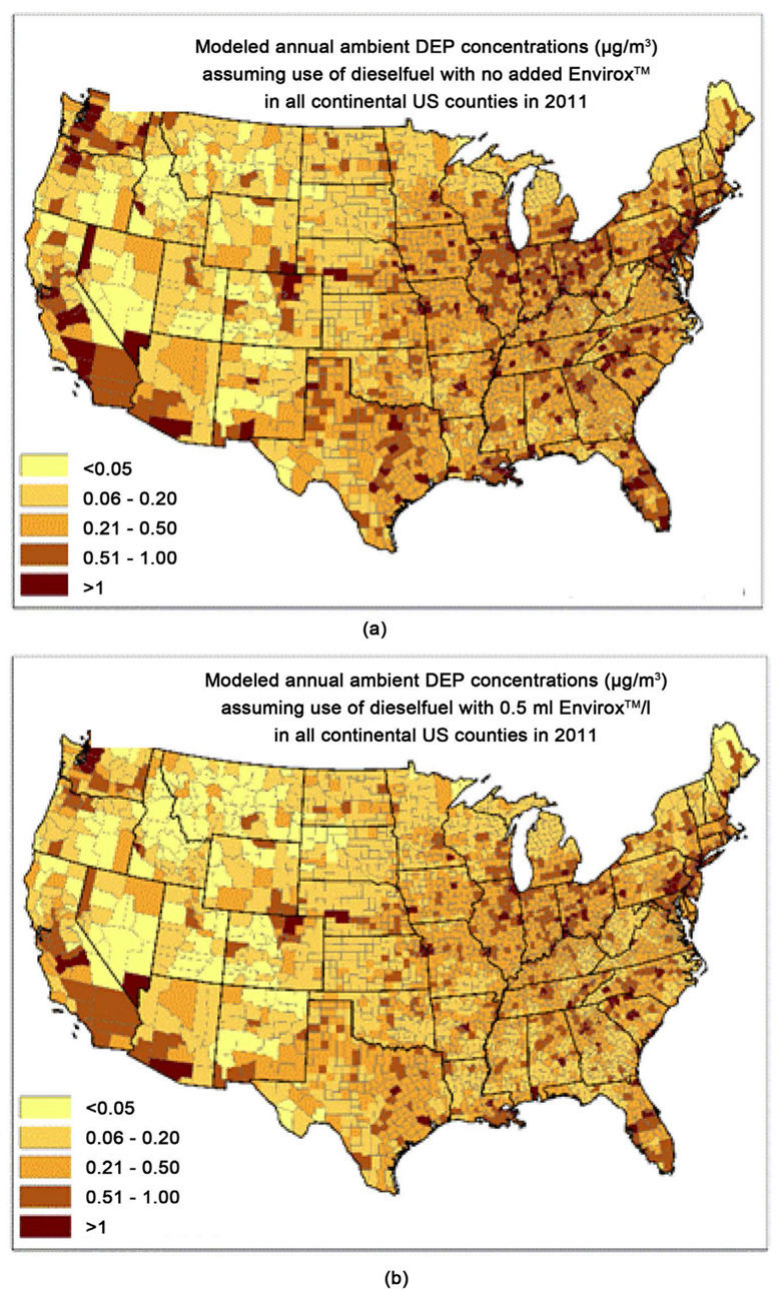
Modelled ambient concentration of diesel exhaust particles (DEPs) assuming the use of diesel fuel with (a) no Envirox^™^ and (b) with 0.5 mL Envirox^™^ added per liter of fuel. The results are based on USEPA’s 2011 National Air Toxics Assessment (NATA) data and the DEPs emission data published in Zhang *et al*., 2013.

Another research focus of the RAMNUC consortium was the investigation of the impact of a nano-ceria diesel fuel additive (Envirox^™^) on pollutant emissions and physicochemical and toxicological properties of diesel exhaust particles (DEPs). Addition of nano-ceria to an ultralow sulphur diesel fuel reduced the emission rates of carbon monoxide, carbon dioxide, formaldehyde, acetaldehyde, acrolein, several polycyclic hydrocarbons, and DEPs mass. However, there was also an increase in the emission rates of nitrogen oxides and ultrafine particles. Modeling studies suggest the nano-ceria additive has the potential to reduce the overall ambient DEPs concentration in many regions of the US (Figure 2). The addition of nano-ceria also affected several physicochemical properties of DEPs (e.g., reducing particle size, reducing carbon content, increasing cerium content, increasing organic carbon to elemental carbon ratio, and reducing oxidation potential) [69] [70]. These transformations may be responsible for the changes in bioreactivity and reduction of DEPs toxicity observed in several experiments conducted with cultured human lung cells, mice cells and zebrafish embryos. Researchers also characterized an effect on immune responses in blood monocytes which was linked to changes in size and zeta potential of DEPs induced by nano-ceria [71].

To capture realistic indoor and outdoor exposure scenarios at the population level, RAMNUC has taken a modular modeling approach including the use of geographic information systems and particle size distributions. A novel tiered modeling system, Prioritization/Ranking of Toxic Exposures with GIS (Geographic Information System) Extension (PRoTEGE), was developed utilizing available data for Ag nanoparticles production, usage, and property databases. The data were complemented with laboratory measurements of potential exposures from Ag nanoparticles-containing consumer spray products generated by RAMNUC [66]. The RAMNUC team developed models of nanoparticle fate, behavior, and *in vitro* toxicity [72] [73] which can be used to support the analysis and prediction of *in vivo* effects by predicting changes in cellular mechanisms caused by the nanoparticles. RAMNUC further investigated the effects of nanoparticle properties on biological toxicity using the Agglomeration-Diffusion-Sedimentation-Reaction Model (ADSRM) and used a direct Monte Carlo simulation to study the transformation of nanoparticles in biological media [72] [73]. Model predictions for agglomeration and dissolution were compared to *in vitro* measurements for various MNMs, coating materials, and incubation media, and found to be consistent with the measurements. The fate, transport, and effects of MNMs throughout the lung were also modelled to predict particle transport across the air/biological fluid interface and the final expected dose for both coated and uncoated particles[73] [74].

## 3. Legacy

The US-UK joint program in nanotechnology research has allowed US and UK scientists to collaborate and share knowledge across national borders in an effort to understand the complexities associated with nanomaterial toxicity and environmental behavior. The international collaboration provided a platform for the consortia to take a robust, systematic approach that pooled strengths and expertise across disciplines. These projects have supported transboundary working practices and allowed the leveraging of additional funding promoting effective use of limited research resources. For example, TINE has developed a joint US-European Union collaboration to optimize the use of nanomaterial-containing products for agricultural benefits. The new project, NanoFARM, is expected to play a key role in ensuring the safe development of agricultural nanotechnology and free trade of agricultural commodities. Additional financial support has been secured from both UK and US funders (e.g. USCPSC, UKNERC, US National Science Foundation, and the Royal Society), with research outputs feeding into broader scientific groups within the nanoscience field. Researchers have developed close communication across organizations, taking advantage of career changes and academic movement between organizations to further build networking opportunities and to grow the research community. Indeed, these difficult to quantify developments may be some of the most important outcomes of this transatlantic program. Notably, the program has substantially advanced the understanding of how nanomaterials enter and move through the environment, how they transform, and how they may affect human health and ecosystems. Researchers have characterized the functional relationship between the nanomaterials physicochemical properties, their toxicity, and effect on organisms, and demonstrated this relationship is amenable to modelling. A key output from this program has been the development of models that characterize the fate of nanomaterials through the environment and predict the impacts they might have on the environment and human health.

Although these collaborations have substantially advanced the knowledgebase, additional work is needed to further progress. The US-UK transatlantic program offers an ideal funding model to enhance progress while effectively making use of scarce resources. An important development is that future funding models should include a more direct link between innovation and risk-related research. These are two cognate areas that are often separated for structural reasons but their interdependency and integration is essential for the safe and sustainable development of nanotechnology. Results reported here point to important research questions that future work will need to address:

1. Are current analytical methodologies suitable for parameterizing and validating models? If not, what further methods can be developed?
2. Can existing models be applied to realistic exposure scenarios? If not, what modifications are required?
3. Can existing models be applied to the next generation of nanomaterials? If not, how can these models be optimized?

## Figures and Tables

**Table 1:** UK-US Nanotechnology consortia funded in 2009 focusing on specific media, research objectives and case studies.

Nanotechnology Consortia	Media	Research Objectives and Case Studies
TINE	Terrestrial	Understanding environmental fate and transformation of MNMs in terrestrial ecosystems Case studies: Transformation in wastewater treatment plants; Life cycle assessment model of terrestrial effects
NanoBee	Aquatic	Understanding exposure, bioavailability, and toxicity of MNMs in aquatic ecosystems Case studies: Transformations and novel methods for MNMs analysis in complex matrices
RAMNUC	Atmospheric, Indoor air	Understanding human toxicity of consumer product-incorporated MNMs Case studies: Household sprays with zinc oxide and silver; Diesel fuel additives with cerium dioxide

The purpose of this paper is to summarize and integrate research results obtained by the three consortia in the transatlantic program.

## Footnotes

1 Comprised of UK NERC, the UK Government Department of Environment, Food, and Rural Affairs, the Environment Agency, Biotechnology and Bioscience Research Council, Engineering and Physical Sciences Research Council, Medical Research Council, and UK Government Department of Health.

## List of Abbreviations

ADSRM: Agglomeration-Diffusion-Sedimentation Reaction-Model
CPSC: Consumer Product Safety Commission
DEPs: Diesel Exhaust Particles
ENI: Environmental Nanoscience Initiative
EPA: Environmental Protection Agency
GIS: Geographic Information System
LCA-RA: Life-Cycle-Analysis-Inspired Risk Assessment
MNMs: Manufactured Nanomaterials
NanoBee: Manufactured Nanomaterial Bioavailability & Environmental Exposure
NNI: National Nanotechnology Initiative
NERC: Natural Environment Research Council
ORD: Office of Research and Development
ProTEGE: Prioritization/Ranking of Toxic Exposures with GIS Extension
RAMNUC: Risk Assessment for Manufactured Nanoparticles Used in Consumer Products
STEM-EELS: Scanning Transmission Electron Microscopy and Electronic Energy Loss Spectroscopy
STAR: Science to Achieve Results
TINE: Transatlantic Initiative for the Nanotechnology and the Environments
ZnONWs: Zinc Oxide Nanowires

## Appendix

https://www.whitehouse.gov/files/documents/ostp/NSTC%20Reports/NNI2000.pdf Leading to the Next Industrial Revolution A Report by the Interagency Working Group on Nanoscience, Engineering and Technology Committee on Technology National Science and Technology Council February 2000 Washington, D.C.

https://royalsociety.org/~/media/Royal_Society_Content/policy/publications/2004/9693.pdf The Royal Society & The Royal Academy of Engineering. 2004. Nanoscience and nanotechnologies: Opportunities and uncertainties.

http://webarchive.nationalarchives.gov.uk/20070603164510/http://www.dti.gov.uk/files/file14873.pdf Response to The Royal Society and Royal Academy of Engineering Report: “Nanoscience and nanotechnologies: opportunities and uncertainties”. By HM Government in Consultation with the Devolved Administrators. 2005.

https://www.gov.uk/government/uploads/system/uploads/attachment_data/file/228871/7468.pdf Novel Materials in the Environment: The case of nanotechnology. Royal Commission on Environmental Pollution. 2008.

https://www.gov.uk/government/uploads/system/uploads/attachment_data/file/228785/7620.pdf UK Government Response to The Royal Commission on Environmental Pollution (RCEP) Report “Novel Materials in the Environment: The Case of Nanotechnology.” 2009.

https://www.epa.gov/research-grants

https://cfpub.epa.gov/ncer_abstracts/index.cfm/fuseaction/display.rfatext/rfa_id/516

http://www.nerc.ac.uk/research/funded/programmes/nanoscience/ao-eni2/
